# Estimating Age of Mature Adults from the Degeneration of the Sternal End of the Clavicle

**DOI:** 10.1002/ajpa.22639

**Published:** 2014-10-18

**Authors:** Ceri G Falys, Dennis Prangle

**Affiliations:** 1 Department of Archaeology, School of Archaeology, Geography and Environmental Science, University of ReadingReading, Berkshire, RG6 6AB, UK; 2 Department of Mathematics and Statistics, Whiteknights, University of ReadingReading, Berkshire, RG6 6AX, UK

**Keywords:** bioarchaeology, elderly, macroscopic bone degeneration, skeletal aging

## Abstract

The sternal end of the clavicle has been illustrated to be useful in aging young adults, however, no studies have investigated what age-related changes occur to the sternal end post epiphyseal fusion. In this study, three morphological features (i.e., surface topography, porosity, and osteophyte formation) were examined and scored using 564 clavicles of individuals of European ancestry (*n* = 318 males; *n* = 246 females), with known ages of 40+ years, from four documented skeletal collections: Hamann-Todd, Pretoria, St. Bride's, and Coimbra. An ordinal scoring method was developed for each of the three traits. Surface topography showed the strongest correlation with age, and composite scores (formed by summing the three separate trait scores) indicated progressive degeneration of the surface with increasing chronological age. Linear regression analyses were performed on the trait scores to produce pooled-sample age estimation equations. Blind tests of the composite score method and regression formulae on 56 individuals, aged 40+ years, from Christ Church Spitalfields, suggest accuracies of 96.4% for both methods.

These preliminary results display the first evidence of the utility of the sternal end of the clavicle in aging older adult individuals. However, in the current format, these criteria should only be applied to individuals already identified as over 40 years in order to refine the age ranges used for advanced age. These findings do suggest the sternal end of the clavicle has potential to aid age estimates beyond the traditional “mature adult” age category (i.e., 46+ years), and provides several suggestions for future research. Am J Phys Anthropol 156:203–214, 2015. © 2014 The Authors American Journal of Physical Anthropology Published by Wiley Periodicals, Inc.

Accurate adult skeletal aging is integral to our understanding of mortality in the past, providing part of an essential biological profile for anthropologists. Age-at-death is also a crucial factor to inform wider archaeological debates about the life course, and the quality and experience of individuals within different life stages and in contrasting societies (Kemkes-Grottenthaler, 2002; Gilchrist, 2004; Wittwer-Backofen et al., 2008). Our ability to accurately estimate the age of older adults has consistently been an area of contention within biological anthropology. Once an assessment has been made, using multifactoral evidence, an estimate of age is stated in a broad range of values to account for the variation of the aging process both within and between individuals. This variation is the result of a multitude of inter-related factors such as genetic disposition, lifestyle (e.g., mechanical stresses), nutrition, health, and chronological age (Crews and Garruto, 1994). The reported maximum (i.e., oldest) open-ended age range for adult individuals varies significantly between researchers and geographical location. In Europe, the most commonly designated age category is 46+ years, with further demarcation rarely identified (Falys and Lewis, 2011). By using a catch-all age bracket of 46+ years for all “older adults”, archaeological age distributions do not accurately represent the record of older-aged adults. This can lead to the assumption that archaeological individuals regularly died at an earlier age than modern humans, despite historical documents, art, sculptures, and gravestones provide evidence to the contrary. This mixture of factors has rendered the “elderly” invisible in the archaeological record (Gilchrist, 2004,2012). It will only be possible to explore the lives and deaths (i.e., social treatment, health, status, longevity, burial, and life course) of the very old from a past perspective when we have the techniques to confidently identify the aged in skeletal assemblages.

Several macroscopic aging techniques do allow for the assessment of age beyond 46 years (e.g., sternal end of the 4th rib, the auricular surface of the ilium, pubic symphysis). Such methods track progressive degenerative changes from the late teens, until the observations reach their peak breakdown and plateau, most commonly between 50 and 60 years (İşcan et al., 1984a,b, 1985; Lovejoy et al., 1985; Brooks and Suchey, 1990; Buckberry and Chamberlain, 2002). While such methods are useful up to the approximate age of 60 years, they do not provide the criteria for further demarcation past this maximum age, resulting in the inability to identify the very old (i.e., 70+ years).

In an attempt to extend the maximum cut-off of adult aging beyond the current limits, research was undertaken to identify new sites of skeletal degeneration that may be proven to display an age-dependent progression (Falys, 2012). This study focuses on the sternal end of the clavicle. As the clavicle does not complete fusion until the late 20s or early 30s (Scheuer and Black, 2004), surface degeneration, if observed, would begin later in adulthood than previously examined areas of the skeleton. The aim was to identify distinct stages of surface breakdown which could be used to develop reliable and reproducible aging criteria, within sufficiently broad age ranges. The ultimate goal was to provide a means to aid in identification the eldest members of a skeletal population within the broader standard category of “mature adult” (i.e., 46+ years).

Previous studies of the human clavicle have investigated the ability to estimate age by radiographic (Walker and Lovejoy, 1985), microscopic (Stout and Paine, 1992; Stout et al., 1996) and destructive macroscopic means (Kaur and Jit, 1990). Such methods are not desirable as they require irreversible damage to the clavicle or specialized equipment and as a result, are not readily applicable. In contrast, nondestructive macroscopic methods derived from the sternal clavicle have a long history of use in skeletal aging, although this has been strictly limited to epiphyseal fusion (Stevenson, 1924; Todd and D'Errico, 1928; McKern and Stewart, 1957; Szilvássy, 1980; Webb and Suchey, 1985; Scheuer and Black, 2004; Meijerman et al., 2007; Cardoso, 2008; Langley-Shirley and Jantz, 2010). All of these studies have identified the importance of the sternal end in providing precise age estimations between the approximate ages of 16 and 30 years, although recently secular changes in the timing of the epiphyseal union of the sternal clavicle have been documented in Americans during the past century (Langley-Shirley and Jantz, 2010; Shirley and Cridlin, 2012). Investigations into the progressive degeneration of the soft tissues of the sternoclavicular joint have been reported in the clinical literature (DePalma, 1957; Arlet and Ficat, 1958; Silberberg et al., 1959; Yood and Goldenberg, 1980; Waterman and Emery, 2002). DePalma (1957) found that degeneration of the fibrous disc covering the sternal end of the clavicle commenced around the fourth decade and continued through the ninth decade of life. Until now, no studies have been carried out mapping changes to the morphology of the sternal end once the epiphysis has fused.

This study was undertaken to assess whether the clinical observations of degeneration of soft tissues of the manubriosternal joint were reflected on the sternal surface of the clavicle itself. An ordinal scoring system was developed to aid in the identification and qualification of degenerative traits on the surface, and as a means to assess the relationship between observed trends in degeneration and chronological age. The findings were then blindly tested on an historic documented skeletal collection to test the accuracy of the proposed method and age estimates.

## Materials

The study sample comprised the left and right clavicles of 564 white individuals, with known age-at-deaths of at least 40 years, from four contrasting documented skeletal collections. Ages ranged from 40 years to 96 years (Table 1). To provide a method that could be applied to many different populations, the skeletal collections were chosen to represent a variety of geographical locations, socio-economic statuses, and time periods.

**Table 1:** Age composition of study samples

Age category (years)	Males					Females				
	HT	PBC	SB	CC	Total	HT	PBC	SB	CC	Total
40−49	10	4	6	20	40	10	1	5	14	30
50−59	18	13	9	22	62	12	4	7	16	39
60−69	19	22	9	21	71	15	16	11	17	59
70−79	24	32	10	21	87	14	24	7	16	61
80+	23	24	4	7	73	14	20	6	17	57
Total	94	95	38	91	318	65	65	36	80	246
Mean age	68.1	70.9	68.7	61.6	66.6	66.1	74.0	65.4	65.5	67.9
Range of ages	42−96	40−94	41−88	40−96	40−96	41−93	48−94	42−91	40−95	40−95

The Hamann-Todd Human Osteological Collection (HT) is an early 20th century U.S. skeletal collection, and is housed at the Cleveland Museum of Natural History, Ohio, USA (Mensforth and Latimer, 1989). The Pretoria Bone Collection (PBC) contains the remains of individuals who died in the late 20^th^ century, and is held in the Department of Anatomy at the University of Pretoria, South Africa (Steyn et al., 2010). The historic St. Bride's Documented Skeletal Collection (SB) dates from the 18th to 19th centuries (Scheuer and Black, 1995), and is still housed in the crypt of St. Bride's Church, Fleet Street, London, UK. Finally, the Coimbra Human Identified Osteological Collection (CC) comprises individuals who were buried during the late 19th and early 20th centuries, and are held in the Anthropology Museum in the Department of Anthropology at the University of Coimbra, Portugal (Cunha, 1995).

## Methods

When present, both clavicles for each individual were examined for three separate traits of degeneration using the scoring system outlined below. The scoring system was developed using established terminology wherever possible, and written descriptions and photographs illustrating each gradient of surface feature were provided to reduce any intra- or inter-observer error. The ordinal scoring system provides a numerical value that reflects the presence and severity of three traits of bone degeneration: surface topography, porosity, and osteophyte formation:

### Surface topography

**Figure 1:**
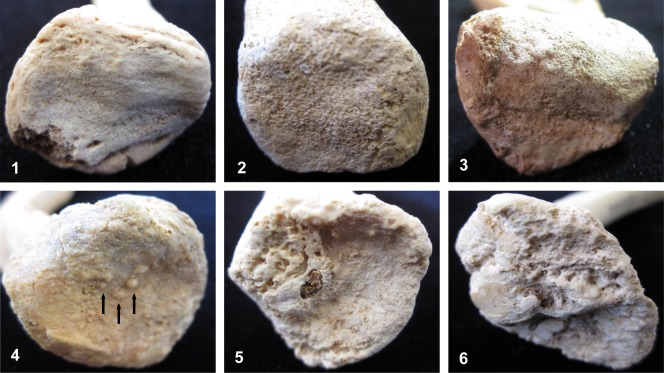
Examples of surface topography trait expression of the sternal surface of the clavicle. Numerical score provided. (1) fusing epiphysis, smooth texture; (2) slight granulation; (3) coarse granulation; (4) nodule formation (arrowed); (5) undulating; (6) surface breakdown. [Color figure can be viewed in the online issue, which is available at http://wileyonlinelibrary.com.]

Surface topography refers to the general texture and relief of the joint surface. Traits examined include the presence of fine or coarse granulation, formation of nodules or marked undulation of the surface. The scoring system for this trait is provided in Table 2, with written descriptions and terminology definitions in Table 3, and photographs for comparison provided in Figure 1.

**Table 2:** Summary of degenerative traits recorded

Score	Topography	Porosity	Osteophyte formation
0	element not present	element not present	element not present
1	smooth	no porosity	no osteophytic growth
2	slight granulation	microporosity (<50% surface)	slight osteophytes
3	coarse granulation	microporosity (>50% surface)	moderate osteophytes
4	nodule formation	macroporosity (<50% surface)	severe osteophytes
5	undulating	macroporosity (>50% surface)	–
6	degeneration/eburnation	complete surface breakdown	–

**Table 3:** Definitions of terminology used for surface topography

Trait expression	Score	Description
Smooth	1	The surface is flat and smooth to the touch. The epiphysis may be in the process of fusing.
Slight granulation	2	The surface texture of the bone is that of fine sand paper (a slightly roughened surface).
Coarse granulation	3	Very small grains of bone form on the surface, resembling coarse sand. The texture is very rough, like sandpaper.
Nodule formation	4	At least one round lump of bone is present on the generally flat surface.
Undulating	5	The topography changes from a smooth and flat surface to an irregular and undulating surface, resulting from development of ridges, severe nodule or osteophyte formation. The outline of the surface also becomes irregular.
Degeneration	6	The surface displays complete breakdown (i.e., is dense, with increased porosity and osteophyte growth) and has highly irregular contours. In many instances, the bone takes on the appearance of honeycomb, with extensive macroporosity across the entire surface.

### Porosity

**Figure 2:**
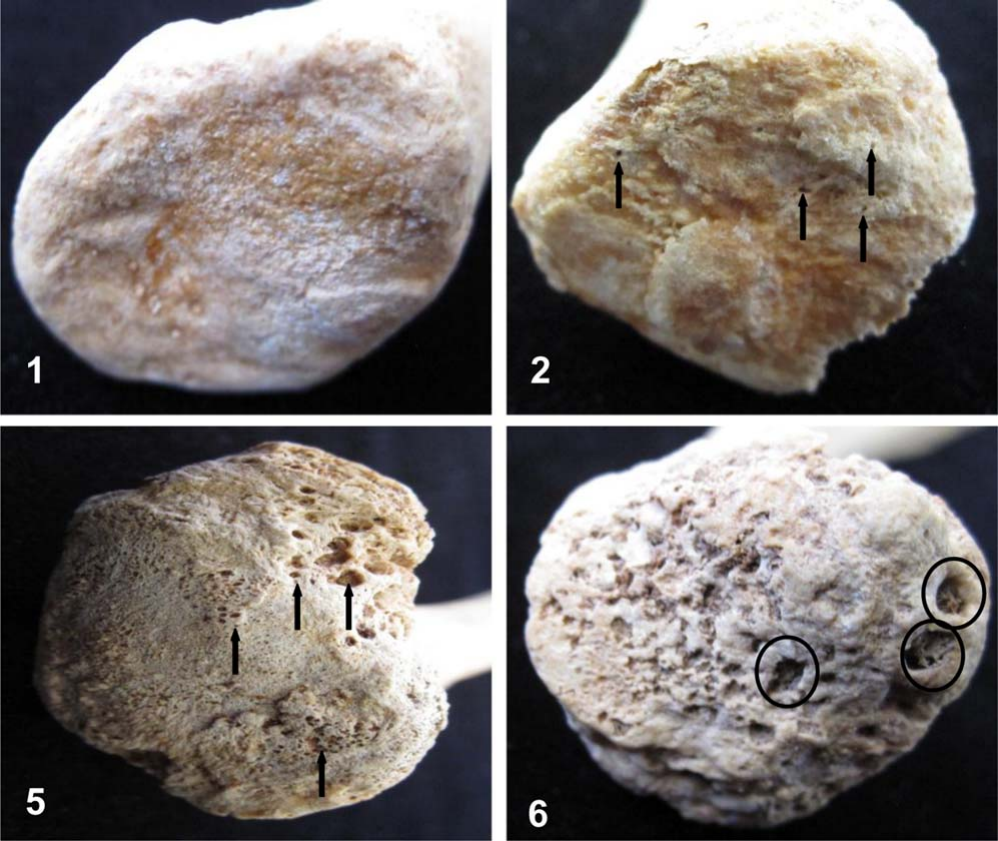
Examples of porosity recorded for the sternal surface of the clavicle. Numerical scores provided. (1) no porosity present; (2) microporosity, dependent on ±50% of surface affected (examples arrowed, score of 2 depicted); (5) macroporosity, dependent on ±50% of surface affected (examples arrowed, score of 5 depicted); (6) converging macroporosities (large erosive surface defects, circled). [Color figure can be viewed in the online issue, which is available at http://wileyonlinelibrary.com.]

The presence and severity of porosity was recorded on a scale indicating no expression (Grade 1) to severe surface defects (Grade 6), outlined and illustrated in Table 2 and Figure 2. Microporosities are defined as very small perforations in the bone surface, measuring less than 1mm in diameter, while macroporosity are large more irregular perforations, greater than 1mm in diameter. The percentage of the surface displaying porosity was also recorded within the system, noting whether the porosity affected more or less than 50% of the sternal clavicular surface. If both types of porosity are present, the percentage of the surface displaying macroporosity is to be recorded. A score of 6 was given if area(s) of deep pitting caused by the convergence of separate macroporosities were present on the surface (circled in Fig. 2, score of 6).

### Osteophyte formation

**Figure 3:**
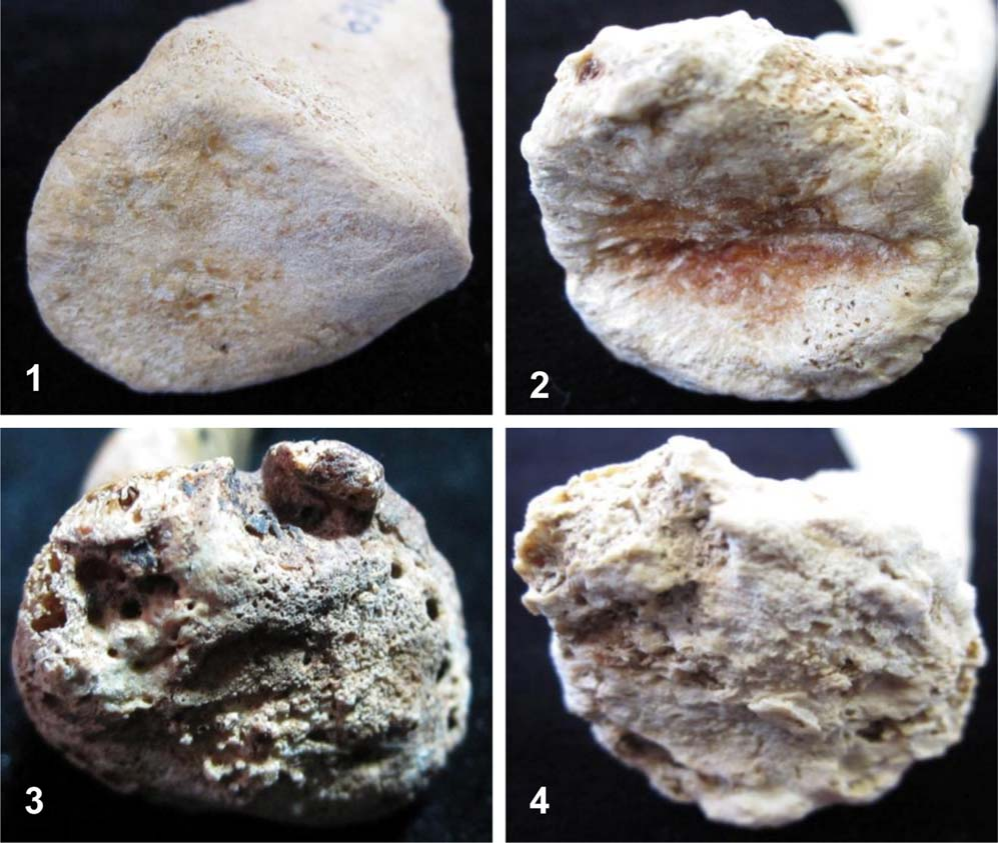
Examples of severity of osteophyte formation recorded on the sternal surface of the clavicle. Numerical scores provided. (1) No osteophytes (smooth surface outline); (2) slight osteophyte formation (roughened outline); (3) moderate expression (definite osseous projections); (4) severe osteophytes (highly irregular surface outline caused by large bone growths). [Color figure can be viewed in the online issue, which is available at http://wileyonlinelibrary.com.]

Osteophyte formation was scored on a four-point scale, from no osteophytes present (Grade 1), to severe osteophytic growth (Grade 4) (see Table 2, Fig. 3). Severity of lipping takes into consideration the generalized osteophyte length and the amount of the clavicular rim that display bony projections. Although it may have made recording easier, a metrically determined osteophyte length was not considered an accurate measure of severity as it fails to take into account the overall size of the sternal end which varied across individuals.

The individual trait scores were then summed to provide a “composite score” for the sternal end of the clavicle, ranging between 3 and 16 points. This composite score was used to assess degenerative differences between the left and right elements, as well as between the sexes. In addition to recording the severity of each trait independently, written descriptions were provided for the overall appearance of joint surfaces that fell within each composite score, with the goal of correlating these with documented age. The aim was to assess whether this progressive surface degeneration could be useful in adult age estimation, and whether the samples could be combined to provide a measure of age applicable to European and European-American populations from different geographical regions. Linear regression analyses were subsequently preformed on the trait scores to produce pooled-sample age estimation equations.

A subsample of 10 males and 10 females from each of the four skeletal collections were re-recorded to test for intraobserver error (*n* = 80). Paired *t*-tests found no significant variation between the trait scores and composite scores recorded during the two analyses (surface topography, *t* = −0.77, *P* = 0.442; porosity, *t* = −0.57, *P* = 0.567; osteophyte formation, *t* = 0.81, *P* = 0.418; composite scores, *t* = −0.90, *P* = 0.369).

The applicability of the developed criteria was also assessed for interobserver error. Ten undergraduate students from the University of Reading, UK, with varying degrees of experience with human skeletal remains, recorded the traits of topography, porosity and osteophyte formation on a sample of 10 individuals, of medieval date, from St. Oswald's Priory, Gloucestershire (Mills, 2013). To assess interobserver agreement Light's kappa (Conger, 1980) was computed. This extends the weighted kappa statistic of Cohen (1968) which is valid for a pair of raters to a multirater setting by taking the mean value over all possible pairs of raters. The values and standard errors, obtained by bootstrapping, were: topography 0.76 (0.10), porosity 0.58 (0.12), osteophyte formation 0.55 (0.10). This shows moderate to strong agreement between observers for all measurements, with more agreement for topography.

### Differences between left and right elements

As degeneration of the shoulder joint may be affected by habitual movement, and hence occupational activity and handedness may effect degeneration, a paired *t*-test was used to compare the resultant composite scores of the left and right sides of all individuals with both clavicles present, to assess whether the recorded states of degeneration were bilateral. No statistically significant differences were identified in trait expression between left and right sides (i.e., *P* > 0.05), with the exception of the CC sample (*t* = −2.24, df = 165, *P* = 0.027). As a result, all subsequent analyses were made using data recorded for the right clavicle, due to this element being most frequently present in the collections, compared to the left side (right clavicles, *n* = 564; left clavicles, *n* = 546). Based on the different result of the CC sample, in this study, the left clavicle was not substituted in absence of the right side.

### Differences between males and females

The presence of sex differences in trait expression was evaluated, as men and women commonly undertake different tasks, occupations, and have been shown to display sexually dimorphic skeletal appearances (Buikstra and Ubelaker, 1994). To avoid low sample sizes, the study samples were pooled. For each value of composite score a *t*-test was performed on age, comparing males and females (Table 4). The results in Table 4 show a tendency for the mean age of female skeletons to be greater for the same combined score. Following a Bonferroni correction for multiple testing, *P*-values show there is a significant difference (*a* = 0.05) for composite scores of 6 and 11. More detailed investigation of the sex effects on age conditional on trait values and the study sample of origin are explored later by model-based methods.

**Table 4:** T-test results comparing age for males and females

Composite score	Observations	Age difference (female–male)		*P*-value	
		Mean	95% CI	Uncorrected	Corrected
4	20	0.07	−7.00,7.14	0.9820	1.0000
5	57	1.67	−2.13,5.46	0.3820	1.0000
6	55	10.5	5.08,15.90	0.0005	0.0054
7	69	5.38	−0.29,11.10	0.0626	0.6880
8	72	1.48	−3.42,6.38	0.5480	1.0000
9	108	5.00	1.37,8.63	0.0074	0.0819
10	71	−0.30	−4.85,4.25	0.0895	1.0000
11	109	4.88	1.57,8.19	0.0044	0.0485
12	30	0.27	−7.620,8.17	0.9410	1.0000
13	13	4.43	−1.24, −10.10	0.1110	1.0000
14	8	−3.75	−15.20,7.70	0.4530	1.0000

Results are omitted for combined scores of 3, 15, and 16 as there were no female observations. *P*-values are shown for a two-tailed test, before and after a Bonferroni correction.

There was no evidence of either sex having significantly more difference between trait expression between the left and right sides of the body. To determine this, each of the three traits (i.e., topography, porosity, osteophyte formation) was considered in turn as follows. The training data was split into those with different scores for the two sides and those with no difference. A statistical test was then performed to determine whether the proportion with differences was the same for both sexes. This test looked at the difference in observed proportions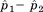 under the null hypothesis that the true proportions are the same. To compute a p-value it was assumed that the test statistic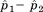 is normally distributed, which is reasonable for the study sample sizes. For all traits, no statistically significant differences were found in any study sample. Pooling the study samples, under a 2-sided test, resulted in *P*-values of 0.77 (topography), 0.95 (porosity) and 0.79 (osteophyte formation).

### Correlation of surface features with known age

All individual trait expressions in all study samples were found to be significantly correlated with age-at-death (Table 5). The higher the score assigned to a particular trait expression, the more frequently it was associated with older age. This correlation was strongest with surface topography for both males and females. The combination of scores for surface topography, porosity and osteophyte formation also produced a significant correlation with documented ages-at-death in both sexes (Table 5). It is noted that in many cases, the Spearman's rank correlation coefficient for surface topography was more highly correlated with advancing age than any of the other traits and even the composite score correlations.

**Table 5:** Summary of Spearman's rank correlation between age and trait expression of features in each of the study samples

Study sample	Sex	Surface topography		Porosity		Osteophyte formation		Composite score	
		*r*_s_	*P*	*r*_s_	*P*	*r*_s_	*P*	*r*_s_	*P*
HT	Male	0.796	<0.001	0.622	<0.001	0.667	<0.001	0.787	<0.001
	Female	0.783	<0.001	0.568	<0.001	0.425	0.003	0.718	<0.001
PBC	Male	0.606	<0.001	0.539	<0.001	0.320	0.017	0.612	<0.001
	Female	0.398	0.006	0.409	0.010	0.458	0.008	0.446	<0.001
SB	Male	0.766	<0.001	0.651	<0.001	0.637	<0.001	0.781	<0.001
	Female	0.731	<0.001	0.723	<0.001	0.625	0.001	0.827	<0.001
CC	Male	0.780	<0.001	0.571	<0.001	0.606	<0.001	0.762	<0.001
	Female	0.671	<0.001	0.575	<0.001	0.497	0.001	0.650	<0.001

**Figure 4:**
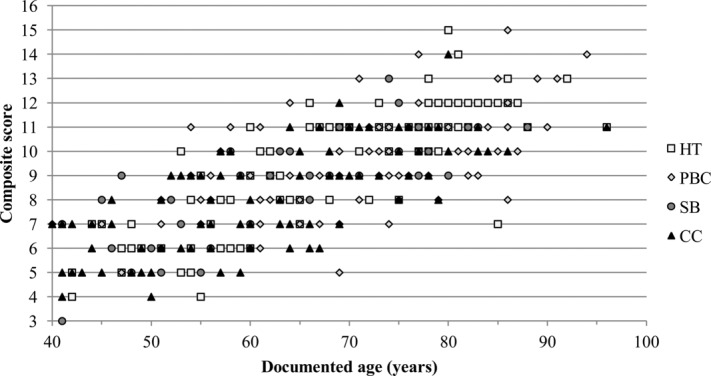
Scatter plot of composite scores against age for males in the combined study sample.

**Figure 5:**
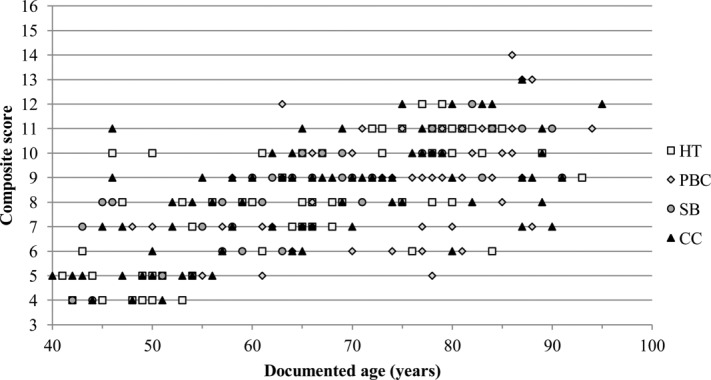
Scatter plot of composite scores against age for females in the combined study sample.

With the exception of the unilateral trait expression in the CC sample, similar general patterns of degeneration were found in all four study samples (i.e., bilateral trait expression, suggested need for sexually dimorphic criteria, and statistically significant correlation between surface feature and age-at-death). Scatter plots of composite scores and known ages for males and females in the combined sample are provided in Figures 4 and 5, respectively. All study samples display similar distributions of increasing composite score with age. To produce potential aging criteria that was nonpopulation specific, the recorded data for all right clavicles were combined for males and females, to assess whether distinct stages of degeneration were present within each age category. These pooled samples comprised 318 male right sternal ends of the clavicle (mean age, 66.6 years) and 246 female clavicles (mean age, 67.9 years) (Table 1).

## Results

### Relationship between trait expression and known age

**Figure 6:**
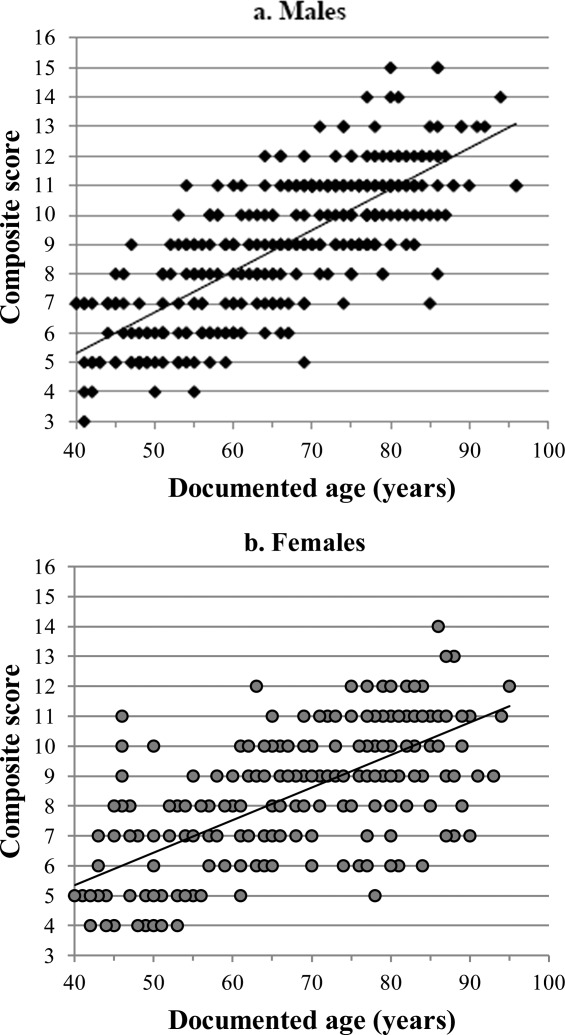
(a,b) Scatter plots of composite scores against documented age for males and females in the combined study sample.

Individual trait expressions were investigated for their association with age-at-death in the pooled samples (Fig. 6). Individual trait expressions were found to be significantly correlated with age-at-death (Table 6). Again, the higher the score assigned to a particular trait expression, the more frequently it was associated with older age. This correlation was strongest with surface topography in both males and females (male, *r*_s_ = 0.762, *P* < 0.001; female, *r*_s_ = 0.665; *P* < 0.001), which was also higher than the relationship between composite score and age (male, *r*_s_ = 0.755; *P* < 0.001; female, *r*_s_ = 0.644; *P* < 0.001).

**Table 6:** Spearman's rank correlation between age and trait expression of features in the combined study sample

Feature	Male		Female	
	*r*_s_	*P*	*r*_s_	*P*
Surface topography	0.762	<0.001	0.665	<0.001
Porosity	0.598	<0.001	0.536	<0.001
Osteophyte formation	0.562	<0.001	0.446	<0.001
Composite score	0.755	<0.001	0.644	<0.001

### Composite score and known age

Tables 7 and 8 provide descriptive statistics for age variation within composite scores for males and females. While the males revealed the overall trend of increasing age with increasing composite score, the picture for females was more complex showing a nonlinear progression of increasing age with composite score.

**Table 7:** Male descriptive statistics per composite score for combined sample (n = 318)

Composite score	*n*	Mean age (years)	Standard deviation	Median age (years)	Observed range (years)
3	1	41.0	−	41	41
4	4	47.0	6.7	46	41–55
5	25	49.4	6.3	48	41–69
6	30	54.9	5.9	55	44–67
7	32	55.4	11.5	55	40–85
8	31	62.8	10.1	63	45–86
9	53	67.3	9.0	68	47–83
10	37	72.5	9.3	75	53–87
11	67	75.2	8.0	75	54–96
12	21	78.1	7.1	80	64–87
13	10	82.9	7.9	85	71–92
14	4	83.0	7.5	80	77–94
15	3	84.0	3.5	86	80–86
16	0	−	−	−	−

**Table 8:** Female descriptive statistics per composite score for combined sample (n = 246)

Composite score	*n*	Mean age (years)	Standard deviation	Median age (years)	Observed range (years)
3	0	−	−	−	−
4	13	47.3	3.6	48	42–53
5	23	50.4	8.4	50	40–78
6	18	66.8	11.4	64	43–84
7	30	62.8	12.2	62	43–90
8	28	64.9	12.0	65	45–89
9	53	71.9	9.8	72	46–93
10	33	72.1	10.1	70	46–89
11	35	79.2	8.5	80	46–94
12	9	79.8	8.5	80	63–95
13	3	87.3	0.6	87	87–88
14	1	86.0	-	86	86
15	0	-	-	-	-
16	0	-	-	-	-

Several consecutive composite scores were found to provide similar age ranges, mean ages, and median ages. Statistical analysis assessed whether the ages displayed by the individual composite scores were significantly different from each other. The consecutive scores that displayed no difference (i.e., *P* > 0.05) were grouped together to form an individual stage (Tables 9 and 10). Five separate stages of degeneration were indicated for both males and females. Each stage was found to be statistically distinct from one another (i.e., *P* < 0.05) (Table 11).

**Table 9:** Descriptive statistics for stages of clavicular degeneration for males (n = 318), based on the combined study sample

Composite score	Age stage	*n*	Mean age (years)	Standard deviation	95% confidence interval (years)
3–5	I	30	48.8	6.3	36–61
6–7	II	62	55.2	9.1	37–73
8–9	III	84	65.7	9.6	47–85
10–12	IV	125	74.9	8.4	58–91
13–16	V	17	83.1	6.9	70–97

**Table 10:** Descriptive statistics for stages of clavicular degeneration in females (n = 246), based on the combined study sample

Composite score	Age stage	*n*	Mean age (years)	Standard deviation	95% confidence interval (years)
3–5	I	36	49.3	7.1	35–63
6–8	II	76	64.5	11.9	41–88
9–10	III	86	72.0	9.9	53–91
11–12	IV	44	79.3	8.4	63–96
13–16	V	4	87.0	0.8	85–89

**Table 11:** Results of t-tests between age stages for males and females in the combined study samples

Stages compared	Male			Female		
	*t*	df	*P*	*t*	df	*P*
I vs. II	−3.91	79	<0.001	−8.45	104	<0.001
II vs. III	−6.69	144	<0.001	−4.36	160	<0.001
III vs. IV	−7.32	207	<0.001	4.19	128	<0.001
IV vs. V	−3.87	140	<0.001	−5.77	45	<0.001

## The Aging Method

In addition to the composite score method, a weighted score was also constructed to predict age, based on the following regression model:

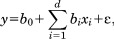
(1)
where y represents age, *x_i_* the *i*th explanatory covariate and *ε* is a random residual. In regression the residual is typically assumed to be normally distributed with location zero and standard deviations. However, this is not appropriate for the training dataset here which consists only of skeletons of age at least 40 years. Therefore, *ε* was assumed to follow a truncated normal distribution with location zero to enforce this constraint. Its standard deviation was considered to take a sex dependent value.

All regression analyses reported here used the same subset of the training data; those for which all the right clavicle measurements were available (*n* = 564). There was no missing data within this subset. This model was fitted to the training data by maximum likelihood. For a similar problem A'Hearn (2004) recommends maximum likelihood incorporating prior knowledge of s, but such knowledge was not available to use this approach here. Residuals of the fitted models were investigated and found to fit the assumed distribution for *ε* well.

Our final choice of model is presented in detail in the results section below. We also considered other choices of which explanatory covariates should be included in the model. Classical methods of model comparison for regressions, such as *F*-tests, are not available for a truncated normal, so instead we used AIC; Akaike's information criterion (for an overview see Hastie et al., 2009 for example). Table 12 presents a selection of results under alternative choices of main effects, and also shows the effect of requiring a single standard deviation of *ε* for both sexes. As well as AIC, the root mean squared error (RMSE) of the model predictions for the blind test data is also given.

**Table 12:** Details of choice of explanatory covariates

Explanatory variables				s.d. of ɛ depends on sex	AIC	RMSE	Estimated ɛ s.d.	
Topography	Porosity	Osteophytes	Sex				Male	Female
*Yes*	*Yes*	*No*	*Yes*	*Yes*	*3972.7*	*8.21*	*8.56*	*10.38*
Yes	Yes	Yes	Yes	Yes	3973.6	8.13	8.54	10.36
Yes	Yes	No	Yes	No	3979.5	8.23	9.41	
Yes	Yes	No	No	No	3999.1	8.09	9.59	
Yes	No	No	Yes	Yes	3996.4	8.82	8.83	10.57
No	Yes	No	Yes	Yes	4259.3	9.15	12.00	12.89

The recommended choice is shown in italics.

We recommend the model with the best fit in terms of AIC in Table 12. Various other models have comparable fits and do slightly better in terms of RMSE. We focus on the AIC score as the training data set is larger and from multiple sites, and so may better indicate how well the results will generalize to future data. The inclusion of information regarding the study sample the clavicle was taken from produced modest improvements to the fit of the statistical model (AIC reduction of up to 3). This shows the relationship between age and the other variables is stable across sites, with some small systematic differences. Interaction terms were also considered. Slight improvements in statistical fit were achieved but as these were very small we opted to present simpler models based on main effects only. It is noted that omitting the osteophyte score produced the best statistical fit than the inclusion of all three trait scores. In Table 12, the recommended choice is shown in italics and the other rows show model fitting results for variations on this choice of covariates. Table 12 is ordered by AIC; lower values indicate better statistical fit. Also shown are the RMSE on the blind test data and estimated standard deviations, to illustrate the amount of residual uncertainty about age under each model fit.

Model (1) makes various assumptions which could be challenged. For example, each score is assumed to have a linear effect. This is likely to be inaccurate, as the scores are ordinal data. However, we found the linear model provides a satisfactory fit to the training data. More specialized ordinal or non-linear regression models are an alternative modelling approach. These are likely to provide improved fits to sufficiently large training datasets, but their predictions may be more difficult to interpret than a simple weighted sum.

Furthermore, (1) assumes that the age is subject to additive measurement error but the covariates are not. Regressions which make this assumption incorrectly are subject to the “calibration problem” (see Aykroyd et al. 1997 for discussion in the context of age estimation), in which covariate effects are underestimated. It may be possible in future work to develop a suitable model for covariate measurement error based on the interobserver error data.

To minimize the biases that can result from the use of regression analyses to estimate age for differing target samples (i.e., “attraction to the middle,” age mimicry of the reference sample), it is recommended that future uses of the current method should be applied to skeletal assemblages with similar demographies as the pooled sample on which these criteria were developed. Details of the how the sample data were selected were given earlier.

The statistical regression analysis produced the following prediction of age:

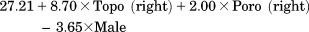


The “male” variable in this equation equals 1 for male individuals (i.e., results in the constant being subtracted), and 0 for females (i.e., the constant is not subtracted). The estimated standard deviation of age around this prediction is 8.56 for males and 10.38 for females. This indicates that there is more uncertainty associated with female age estimation. For the case where sex is not available, an alternative prediction is:

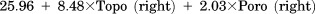


The standard error is 9.59. The regression results allow us to consider the question of whether there are differences in age between male and female skeletons given that the trait scores are the same. The results above indicate that in this situation female skeletons are on average 3.7 years older (from the regression coefficients), and this is a significant effect (Table 12 shows that removing the sex covariate from the model gives a significantly poorer fit). We also investigated whether this result held when considering data from a single site only. Given the same trait scores, female skeletons were estimated to be older by 2.4 years (HT), 4.6 years (PBC), 4.0 years (SB) or 3.4 years (CC). In three cases model fit was improved by including sex and for SB the results were narrowly in favour of removing it (AIC difference of 0.5).

### Blind test of the methods

A blind test was undertaken to determine the applicability and accuracy of both the composite score method and the age estimation formula, using a randomly selected sample of individuals from the Christ Church, Spitalfields Documented Skeletal Collection, housed at the Natural History Museum, London, UK. A total of 56 individuals comprised the blind test sample, all of whom had the sternal end of the right clavicle present for analysis. Although the individuals were identified as 40+ years, the exact age of the sample was not known prior to analysis. The sample comprised 28 males with age-at-death ranging from 40 to 91 years (mean age, 64.3 years) and 28 females, ranging between 44 and 89 years (mean age, 64.8 years).

Of the 56 individuals assessed, the application of the composite score method found 96.4% of both males and females fell within 95% confidence interval. One male (3.6%), aged 88 years old, was underaged by the resultant composite score, and one female (3.6%), who was 78 years old, was overaged. In comparison, the results of the regression equation found the estimated age of two individuals (i.e., 3.6%; one male and one female) fell outside the 95% confidence intervals. A bias towards underaging was suggested, as both of these individuals had an estimated age less than their known ages-at-death.

## Discussion and Conclusions

The current findings correlate well with those of DePalma (1957), who noted that the sternal articular disc begins to increase in thickness in the fourth decade, and continues to increase with advancing age. It has been proposed that this thickening delayed sternoclavicular joint degeneration until the eighth decade, at which time maximum degeneration was reached (DePalma, 1957). Here, peak degeneration was recorded in male individuals over the approximate age of 70 years, and 85 years in females.

This preliminary study is the first of its kind to identify the progressive degeneration of the sternal end of the human clavicle with advancing chronological age; however, more work is needed before this aging criteria can be applied to a wide range of unknown skeletal individuals. At present, the criteria do not include younger individuals aged from the moment of epiphyseal fusion, and to be used, the individual under study would have to be known to be over the age of 40 years. The role of occupation and handedness in the degeneration of this joint also need to be investigated. Although it appears to be stable in three of the four populations assessed in this study, the side differences observed in the Coimbra sample may indicate these issues are important factors. The effect of secular change in the timing of the epiphyseal union and degree of degeneration should also be investigated, as the former has been shown to occur in the Hamann-Todd population (Shirley and Cridlin, 2012).

Well established documented age skeletal samples were used to identify the degenerative trends presented. Although each individual included in this study were stated to have known age, the accuracy of the “known ages” within the Hamann-Todd collection have been questioned (Todd, 1920; Cox, 2000). It is noted, however, the degenerative patterns in this sample did not differ greatly from the three other study samples.

Although the criteria were purposefully produced employing descriptive language frequently used in established aging methods, and photographs were provided for reference, as is always true with aging techniques, the accuracy and reliability of any method is ultimately dependent on the overall experience of the observer. Aging criteria are interpreted and subjectively applied based on the practitioner's experience with skeletal remains and their familiarity with the practical application of the technique. The findings of the interobserver error testing suggest the identification of three dimensional traits using only written descriptions and two dimensional photographs for comparison may be inadequate. While a strong agreement between observers was found for identifying changes in surface topography, the moderate agreement found for porosity and osteophyte formation may indicate that better descriptions or photographs are required for reliable technique application, or perhaps the production of three dimensional casts may be beneficial to ease trait identification.

The 95% confidence intervals for the composite score method and the regression equations for this 40+ year old study group are broad, and as a result, do not provide the essential precision for application to forensic contexts. It is suggested, however, the developed criteria can provide lower age limits for older individuals in forensic settings. It is also noted that one benefit the production of the regression formula is that provides the ability to use the criteria if sex is unknown, which will be an asset for analyzing archaeological remains.

The recent use of improved statistical approaches in combination with established osteological criteria have shown that it is possible to obtain age estimations with much better accuracy than reliance on the original methods alone. Future revisions of this developed criteria may prove that the application of Bayesian methods (e.g., Hoppa and Vaupel, 2002; Prince et al., 2008; Langley-Shirley and Jantz, 2010; Konigsberg and Frankenberg, 2013), or a more probabilistic approach such as transition analysis (Boldsen et al., 2002; Milner and Boldsen, 2012) may provide more accurate and precise estimations of age than presented in this study.

The aims of this project were to assess morphological changes to the sternal end of the clavicle once the epiphysis was fully fused, and to determine if this surface could provide a new way to differentiate “the very old” from within the traditional 46+ year age category. This study has shown that, in the studied samples, the sternal end of the clavicle gradually deteriorates from a generally smooth surface over time. Five progressive stages of degeneration for males and females were identified, which illustrate the trait expression was cumulative with advancing age. Regression analysis indicated that osteophyte formation contributed little to the estimation of age, and the developed formula with new weighted scores for topography and porosity can be used to estimate age for skeletal individuals known to be over the age of 40 years. Although the 95% confidence intervals are broad, the sternal end of the clavicle has shown great promise in the identification of “the elderly” in archaeological skeletal populations, and provides many avenues of future research for both biological anthropology and social archaeology.
